# Combining phase images from array coils using a short echo time reference scan (COMPOSER)

**DOI:** 10.1002/mrm.26093

**Published:** 2015-12-29

**Authors:** Simon Daniel Robinson, Barbara Dymerska, Wolfgang Bogner, Markus Barth, Olgica Zaric, Sigrun Goluch, Günther Grabner, Xeni Deligianni, Oliver Bieri, Siegfried Trattnig

**Affiliations:** ^1^ High Field Magnetic Resonance CentreMedical University of ViennaAustria; ^2^ Department of Biomedical Imaging and Image‐guided TherapyMedical University of ViennaAustria; ^3^ The University of Queensland, Centre for Advanced ImagingBrisbaneAustralia; ^4^ Center for Medical Physics and Biomedical EngineeringMedical University of ViennaAustria; ^5^ Department of RadiologyDivision of Radiological PhysicsUniversity of Basel HospitalBaselSwitzerland; ^6^ Department of Biomedical EngineeringUniversity of BaselSwitzerland

**Keywords:** phase combination, phase imaging, phased array coils, ultra‐high field, parallel transmit

## Abstract

**Purpose:**

To develop a simple method for combining phase images from multichannel coils that does not require a reference coil and does not entail phase unwrapping, fitting or iterative procedures.

**Theory and Methods:**

At very short echo time, the phase measured with each coil of an array approximates to the phase offset to which the image from that coil is subject. Subtracting this information from the phase of the scan of interest matches the phases from the coils, allowing them to be combined. The effectiveness of this approach is quantified in the brain, calf and breast with coils of diverse designs.

**Results:**

The quality of phase matching between coil elements was close to 100% with all coils assessed even in regions of low signal. This method of phase combination was similar in effectiveness to the Roemer method (which needs a reference coil) and was superior to the rival reference‐coil‐free approaches tested.

**Conclusion:**

The proposed approach—COMbining Phase data using a Short Echo‐time Reference scan (COMPOSER)—is a simple and effective approach to reconstructing phase images from multichannel coils. It requires little additional scan time, is compatible with parallel imaging and is applicable to all coils, independent of configuration. Magn Reson Med 77:318–327, 2017. © 2015 The Authors Magnetic Resonance in Medicine published by Wiley Periodicals, Inc. on behalf of International Society for Magnetic Resonance in Medicine

## INTRODUCTION

Phase information is used in susceptibility‐weighted imaging (SWI) 1, quantitative susceptibility mapping (QSM) 2, 3, and susceptibility tensor imaging 4, for the depiction of iron accumulation in neurodegenerative disorders 5 and for mapping in vivo conductivity 6. It can also be used to measure changes in temperature 7 and encode flow velocity in phase contrast angiography 8. Phased array radiofrequency (RF) coils provide higher signal to noise ratio (SNR) than volume coils 9, allow acceleration through parallel imaging and simultaneous multislice methods 10, 11, 12, 13, and enable control over patterns of transmit RF (B_1_^+^) via parallel transmit excitation 14, 15. These features are particularly advantageous at ultra‐high static magnetic field (7 T and above) because of inhomogeneous B1.

Data from array coil elements are optimally combined by weighting each signal by the respective coils' complex sensitivity 16. Coil sensitivities can be determined using a homogeneous reference scan 9, 10. At 3 T and lower field strengths, this is generally acquired with a volume coil, such as a body coil. In the absence of a body coil at ultra‐high field, a local transceive (eg, birdcage) coil may be used, although this may not be very homogenous and may not be engineered to receive signal. This is often the case with parallel transmit arrays, for instance. If sensitivity maps cannot be acquired, the magnitude image from each coil is a reasonable approximation to the magnitude of the sensitivity of that element, and a root sum of squares (rSOS) reconstruction is an SNR‐optimized and computationally efficient method to create a combined magnitude image 17. Combining phase information from the receive array is more challenging, however, since, in addition to susceptibility effects, the measured phase in each coil is subject to a unique *phase offset*.

In reconstructing multi‐echo data, the temporal evolution of the phase can be used to isolate the susceptibility‐related contribution, the usual quantity of interest. Building on phase difference imaging 18, a number of recent methods pose sophisticated, low‐noise solutions to the multi‐echo problem (eg, using singular valued decomposition 19 and the methods known as MAGPI 20 and CAMPUS 21).

The reconstruction of single‐echo data, however, requires phase offsets to be measured, modeled or approximated. Constant contributions to the phase offset can be removed by setting the phase to zero in all coils at the center of the image 22, although the effectiveness of this approach decreases with the distance from the center, and SNR in the combined image decreases accordingly. An alternative solution is to refer single‐channel phases to a *virtual body coil* 23, 24 or *virtual reference coil* 25. This yields excellent phase matching where a combined virtual coil image without signal voids can be generated via a linear combination of the individual channels. This requires overlap in coil signals and can fail for large objects (eg, at circa half a wavelength from the matching position for the method presented in 25), and the combined image retains arbitrary contributions to the phase; that is, it does not reflect magnetic susceptibility alone. Physical phase offsets can be calculated explicitly from a multi‐echo acquisition (MCPC‐3D) 26, although this involves phase unwrapping, which is time consuming and prone to error.

A recent study has shown that a measurement at a short echo time (TE) can be used to phase spectra from array coils 27. We propose a method of combining images from array coils that applies a similar approach to imaging. A reference scan is acquired at an echo time that is sufficiently short to ensure that the phase measured in each coil approximates the phase offset. The phase of this short TE reference image is subtracted from the phase of the scan to be reconstructed, matching the phases in the individual coils, thereby allowing them to be combined. We call this approach *COMbining Phase data using a Short Echo time Reference scan*, or COMPOSER.

## THEORY

The combination of magnitude images is considered first. In the absence of knowledge of coil sensitivities, a combined magnitude image can be generated from the sum of *l* individual channel magnitudes of the target scan (the scan to be reconstructed), $M_{{}_{TARGET,l}}$, as follows:

(1) $$M_{SS}=\sum_{l}M_{{}_{TARGET,l}}.$$

The subscript SS denotes a *simple sum*. As in subsequent descriptions, spatial indices have been omitted as the formalism applies to all voxels equally. An SNR‐optimized combined magnitude image can be generated by weighting the magnitude from each channel by itself (as an estimate of the magnitude sensitivity) in a root sum of squares (rSOS) 17:

(2) $$M_{rSOS}=\sqrt{\sum_{l}M_{{}_{TARGET,l}}^{2}}.$$

Analogous operations cannot be applied usefully to the phase because of disparate phase offsets. The phase offset consists of inhomogeneous B_1_^+^ and B_1_^−^ phase 26, 28, a linear gradient in the readout direction introduced by timing errors in the acquisition 27 and a constant term reflecting eg, the length of the receiver chain. In contrast to the phase arising from local field effects, the phase offset is constant over time.

Neglecting small nonlinear effects caused by multiple water compartments and white matter anisotropy 29, incompletely compensated flow, as well as wraps and noise, the total measured phase *θ_l_* in the *l*th coil at *TE*, is

(3) $$\theta_{l}=2\pi \gamma \Delta B_{0}T_{E}+\theta_{0,l},$$

where Δ*B*_0_ is the local deviation from the static magnetic field, γ is the gyromagnetic ratio, and $\theta_{0,l}$ is the phase offset. When the assumption of linearity holds, a multi‐echo acquisition allows $\theta_{0,l}$ to be calculated from two or more measurements of $\theta_{l}$, although these need to be unwrapped 26.

The simple observation underlying the coil combination with the method proposed here is that $\theta_{l}$ approximates to $\theta_{0,l}$ if *TE* is sufficiently small. We call this phase value *θ_SER,l_*, the phase in a short echo time reference scan (SER). Subtracting *θ_SER,l_* from the phases of the corresponding coils in the scan to be reconstructed, *θ_TARGET,l_*, phase‐matches the coil elements, allowing them to be combined using the complex sum

(4) $$\theta_{COMPOSER}=∠\sum_{l}M_{{}_{TARGET,l}}\times e^{i(\theta_{TARGET,l}-\theta_{SER,l})}.$$

The ∠ symbol denotes the four‐quadrant tangent inverse of the complex sum (which is usually called atan2 in computer languages). Weighting by the square of the magnitudes, rather than the simple magnitude, yields a combined image with noise variation similar to that obtained using the optimum combination if there is no noise correlation between channels 30. We use simple magnitude weighting in this study and neglect noise correlation to allow comparison with other recent methods 22, 26, 31.

Combined magnitude images can also be generated from complex images that are phase‐matched in this way. As an alternative to Eq. (1), a combined magnitude image can be generated from the phase‐matched complex summed signal as follows:

(5) $$M_{COMPOSER\_S}=abs\left(\sum_{l}M_{{}_{TARGET,l}}\times e^{i(\theta_{TARGET,l}-\theta_{SER,l})}\right).$$

SNR is increased by squaring the magnitude images of the target scan to provide sensitivity weighting, analogous to *M_rSOS_*

(6) $$M_{COMPOSER\_W}=\sqrt{abs\left(\sum_{l}M_{{}_{TARGET,l}}^{2}\times e^{i(\theta_{TARGET,l}-\theta_{SER,l})}\right)},$$

in which the subscripts of *M_COMPOSER_* “_*S*” and “_*W*” in Eqs. (5) and (6) stand for *simple* and *weighted*, respectively. The attributes of these phase and magnitude images will be assessed in the following sections. Finally, for high SNR voxels, the quality of phase matching is reflected by the constant Q:

(7) $$Q=100\times \left(\frac{M_{\left[METHOD\right]\_S}}{M_{SS}}\right),$$

where $M_{\left[METHOD\right]\_S}$ is the simple combined magnitude image for the phase reconstruction method being assessed (in the case of COMPOSER, described by Eq. (5)).

Supporting Figure S1 illustrates how the quality of phase matching is reflected in the magnitude ratio Q, which has been used in previous work 26, 32.

## METHODS

Eight healthy volunteers (two females, six males), participated in the study. The six males (age range 25–33 years) took part in the main study of the brain; one healthy female, aged 25, participated in the breast study; and one healthy 23‐year‐old female participated in the calf measurements. The subjects participated with written informed consent to the studies, which were approved by the Ethics Committee of the Medical University of Vienna.

Measurements were made with a 7T MR whole body Siemens Magnetom scanner (Siemens Healthcare, Erlangen, Germany).

The following measurements were performed to allow the comparison of the performance of COMPOSER with other phase combination methods in the brain. Six subjects were studied with a 32‐channel head coil (Nova Medical, Wilmington, Massachusetts, USA), consisting of a birdcage transceive coil and 32 receive elements.

### Target Scan

The scan to be reconstructed was a high‐resolution, axial, three‐dimensional (3D) flow‐compensated gradient‐echo acquisition with TE = 15 ms and repetition time (TR) = 28 ms, GRAPPA factor 2 with a 704 × 572×96 matrix, 0.3 mm in‐plane resolution and 1.2 mm‐thick slices, with a receiver bandwidth of 140 Hz/pixel and acquisition time (TA) = 10 min 18 s. This is a similar protocol to that used in a previous phase combination study 26 and clinical research by our group (eg, 33).

### Short Echo Time Reference Scan

The short‐echo‐time reference scan was acquired with a 3D variable echo time (vTE) sequence. This Fourier‐encoded spoiled gradient‐echo sequence achieves short variable echo times by using nonselective excitation, asymmetric readouts, and the shortest TE possible for each readout 34, 35. The matrix size of the SER scan was 128 × 104×72 (2 × 2×4 mm^3^ resolution), vTE/TR = 0.8/5 ms, receiver bandwidth of 400 Hz/pixel, TA = 11 s.

### Other Reference Scans

A two‐dimensional (2D) dual‐echo gradient‐echo scan was acquired with a monopolar readout and (TE1,TE2)/TR = (4.6, 9.3)/606 ms, GRAPPA 4, TA = 27 s, with a 128x128 matrix and 32 slices of 3.0 mm thickness and a 230x230 FoV (giving 1.7 mm in‐plane resolution) for one of the comparison methods, MCPC‐3D 26.

For one subject, two GE scans were acquired, the first with the 32‐channel phased array, the second with the birdcage transceiver coil, for the Roemer/SENSE method. The parameters were the same as for the MCPC‐3D scan except that they were single echo with TE/TR = 5.0/360 ms.

### Multi‐echo Acquisition

A high‐resolution multi‐echo scan was also acquired to allow comparison between COMPOSER and the phase difference (fieldmap) method 18. This was a triple‐echo gradient‐echo acquisition with a matrix size of 448 × 364×224 (isometric voxels of 0.5 mm side length), monopolar readout and TE = [7.5, 13.0, 19.5] ms. The bandwidth was 310 Hz/pixel, TA = 12 min 3 s.

COMPOSER was also tested with two additional RF arrays, for the breast and calf. In contrast to the head array, these were not able to generate an independent, volume‐reference image with which to perform a Roemer el al. style reconstruction 9.

### Breast Measurements

Measurements of the breasts of a healthy 25‐year‐old female were made with a four‐channel double‐tuned ^31^P/^1^H coil (Stark Contrast; MR Imaging Coils Research, Erlangen, Germany). This coil is optimized for phosphor investigations, but only the proton capability was used in this study. The same two proton coil elements (one for each breast) were used for excitation and reception; however, for reception, the RF signal from each loop was preamplified and processed separately. The target scan was an axial 3D GE acquisition with a matrix size of 192 × 132×160 (1.6 × 1.6 × 1.3 mm^3^ resolution), flip angle (FA) = 5 °, TE/TR = 3.2/7 ms, TA = 1 min 42 s. The SER scan was an axially acquired vTE with a matrix size of 128 × 88×52 (resolution 2.5 × 2.5 × 5.0 mm^3^), FA=5 °, vTE/TR = 0.6/2.3 ms, TA = 11 s.

### Calf Measurements

Measurements of the right calf of a healthy 23‐year‐old female were made with the two‐channel ^1^H array of an RF coil, which is typically used together with a 3 channel ^31^P array to study phosphor metabolism 36. The ^31^P array was removed for these measurements to reduce unnecessary losses resulting from coupling. The two ^1^H channels were decoupled using a shared conductor and capacitor. The RF signal was split into two equal parts via a 90 ° hybrid coupler, simultaneously achieving the 90 ° phase shift needed for quadrature. The target scan was an axial 3D GE acquisition covering the gastrocnemius and soleus muscles, with a matrix size of 256 × 256×120 (resolution 0.55 × 0.55 × 2.0 mm^3^), FA=5, TE/TR = 10.0/15 ms, GRAPPA factor 2, TA = 1 min 41 s. The SER scan was an axial‐acquired vTE with a matrix size of 96 × 96×120 (resolution 1.5 × 1.5 × 2.0 mm^3^), FA=5 °, vTE/TR = 0.6/2.5 ms, TA = 11 s.

The phase and magnitude data for each channel were stored for all acquisitions.

### Analysis

**Figure 1:**
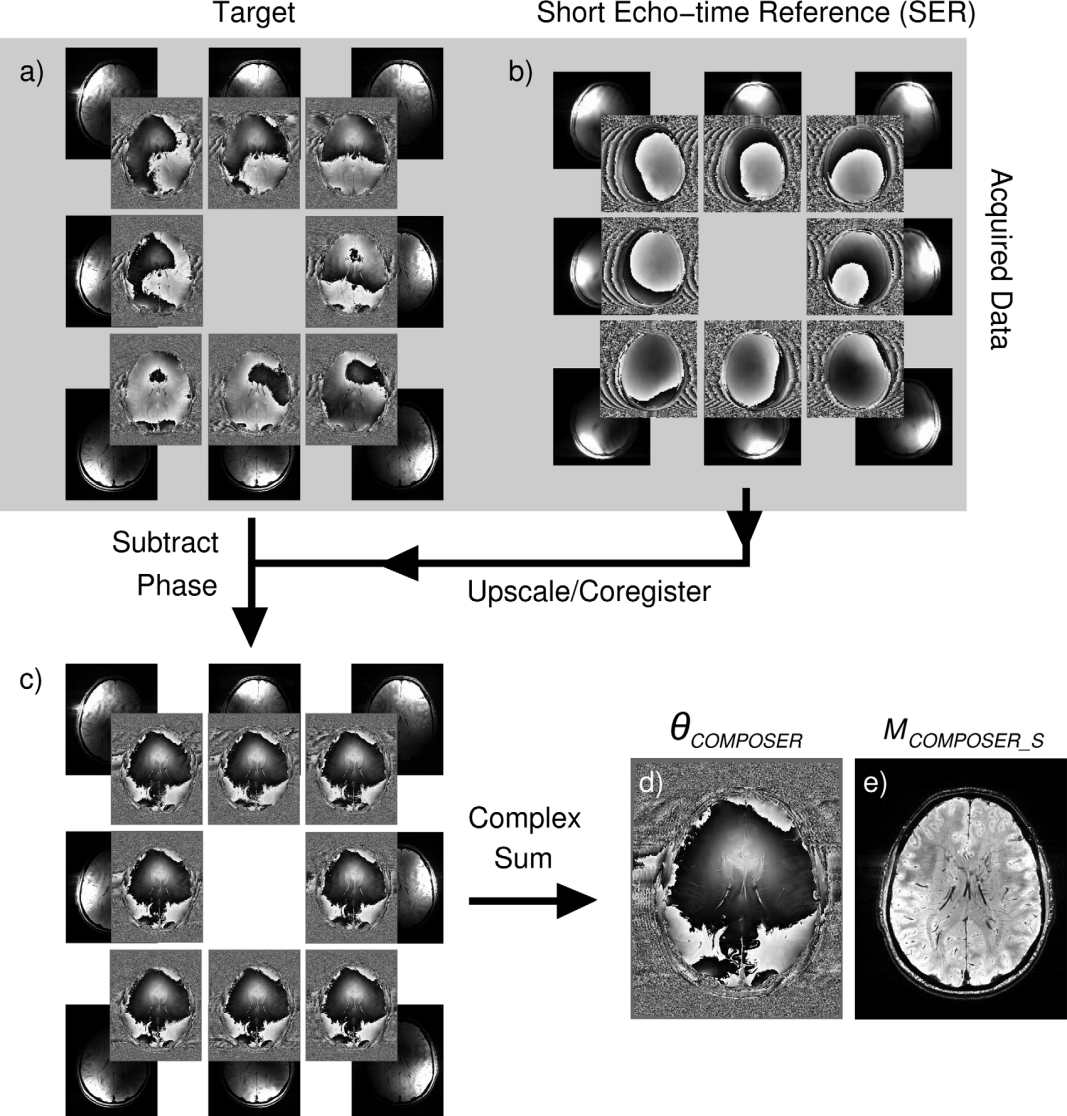
Steps in the COMPOSER method, illustrated for eight channels of a 32‐channel head coil. Single‐channel phase images from the scan to be reconstructed (a) the target—a high‐resolution GRE acquisition—show disparities resulting from surface coil sensitivities as well as common variation due to local deviation from the static magnetic field (eg, in frontal regions). Phase images from a short‐echo‐time reference scan (b) reflect phase offsets; the superposition of a circularly symmetric variation from B_1_^+^ (the same in all images) and a channel‐specific B_1_^−^ are apparent, but no visible susceptibility‐related contribution. SER images are coregistered to the high‐resolution GRE scan, and the SER phase subtracted from the phase of the target scan, after which these appear identical (c). Combined phase (d) and magnitude images (e) are generated according to Eqs. (4) and (5).

Other than the Adaptive Combined reconstruction, which was performed on the scanner's image reconstruction computer, the methods were implemented in and assessed with MATLAB (Mathworks Inc, Natick, Massachusetts, USA). The steps in COMPOSER are illustrated in Figure 1. The SER reference data were coregistered to the high‐resolution scan using FSL's FLIRT (www.fmrib.ox.ac.uk/fsl/) 37. The transformation from the space of the SER scan to that of the target scan was derived from coregistering the SER rSOS magnitude image to the target rSOS magnitude image. This transformation was applied to the single‐channel SER data in rectangular coordinate (real and imaginary) form to avoid artifacts interpolating phase values close to wraps. The SER phase in the target space was subtracted, voxel‐wise and channel‐wise, from the phase of the target scan before the summation over channels and calculation of the combined phase and magnitude according to Eqs. (4) and (5), respectively.

COMPOSER reconstructions were compared with the reconstructions from the following methods: 1) the complex sum of coil signals with no phase correction applied; 2) the Hammond method (an image‐based phase‐matching approach based on the subtraction of a channel‐dependent constant 22); 3) the Adaptive Combined method implemented on the scanner console (Syngo baseline version N4_VB17A_LATEST_20090307); 4) MCPC‐3D‐II 26, which calculates the phase offsets from a dual‐echo reference scan (using PRELUDE in 2D 38 to spatially unwrap separate channel data and interslice phase‐jump corrected as described in 26); 5) the Roemer method 9 (one subject only); and 6) the phase difference method (ie, the voxel‐by‐voxel Hermitian inner product (Eq. (3) in 39; see also 40; applied to the multi‐echo data from one subject).

The quality of phase matching in each voxel was assessed via the metric Q (see Eq. (7)). Histograms of Q were generated within individual brain masks generated with BET (http://fsl.fmrib.ox.ac.uk/fsl/fslwiki/BET) 41. To avoid the inclusion of voxels within the brain which had very low SNR (eg, in large veins), voxels in which the sum magnitude of the target scan was less than 3% of the median were excluded.

Combined phase images were unwrapped with PRELUDE in 2D 38 and the Cusack method 42, and the results were assessed for gross unwrapping errors. To allow a qualitative assessment of phase images generated with approaches 1–5 above, phase wraps were removed from the combined images using Laplacian unwrapping 4 as implemented in STI suite (http://people.duke.edu/~cl160/) and were high‐pass filtered. In the first subject scanned, magnitude images M_SS_, M_rSOS_, M_COMPOSER_S_, and M_COMPOSER_W_ were calculated according to Eqs. (1), (2), (5), and (6), respectively, and noise was estimated using the standard deviation in a background region.

## RESULTS

**Figure 2:**
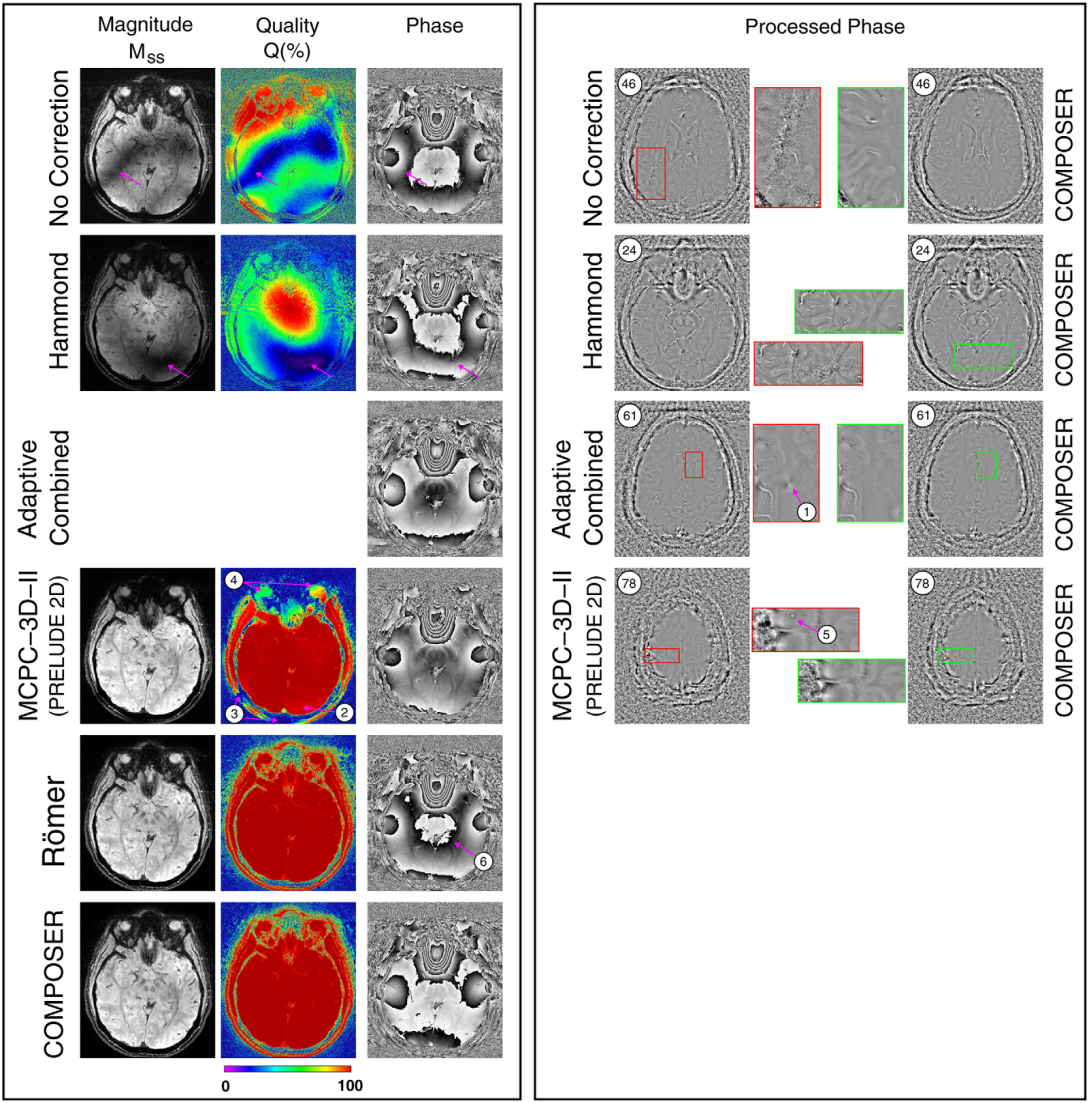
A comparison of the quality of phase images generated with COMPOSER and the other approaches tested (one subject). The left panel shows the same slice (slice 26). Magnitude images (M_SS_) are scaled identically and the quality parameter Q is defined in Eq. (7) (low Q values reveal poor phase matching). No Correction: The magnitude and Q values were generally low and many slices had regions of very low SNR (see slice 46 in right panel). Hammond: The magnitude and Q values were high at the center of the image but low in distal slices, as illustrated in the slice in the right panel. Adaptive Combined: No magnitude or Q maps were available, but phase images showed open‐ended fringe lines, indicating complete signal loss. After processing, these persisted as isolated point artifacts (see arrow 1). MCPC‐3D‐II: Phase matching was generally very good, with most regions having Q values close to 100%. The origin of isolated artifacts (at arrows) is described in the main text and Supporting Figure S3. With both the Roemer method and COMPOSER, the Q values were close to 100% throughout the brain and in regions of disconnected tissue such as the eyes and scalp. No artifacts were apparent in either approach, although there was a larger background phase variation in the Roemer reconstruction.

Reconstructions with all of the single‐echo reconstruction methods under consideration are illustrated for the same subject and slice in Figure 2, left panel. Regions particularly affected by artifacts are shown in the right panel, with enlargements, and compared with the COMPOSER reconstruction of the same slice and enlarged region. The magnitude (Mss) and Q values were low for No Correction and for the Hammond method distal from the center of the image, reflecting poor phase matching (see arrows). The value of Q was approximately 5–10% at the positions marked by arrows, but no open‐ended field lines were visible in the combined phase images. These could be unwrapped with PRELUDE without errors, but were noisy at positions where Q was low, as is apparent in the processed phase images of the other slices (see enlargements in right panel for slices 46 and 24, respectively). No Q values could be calculated for the Adaptive Combined method as a result of a scanner software restriction (that separate channel data could not be written out), but open‐ended fringe lines were evident in some slices, indicating complete signal cancellation. The point artifact shown in one such slice (slice 61, arrow 1) extended over many slices. Generally, magnitude and Q values were high with MCPC‐3D‐II, although errors in unwrapping single‐channel phase values led to reduced Q values within the brain (arrow number 2) and areas of disconnected signal (eg, the scalp, at arrow number 3, the eyes at arrow number 4) and in frontal and ventral areas (in more inferior slices, not shown). An artifact arising from a phase‐unwrapping error is shown in a superior slice, number 78 (arrow 5). Phase matching was almost perfect with the Roemer method and with COMPOSER; images were artifact‐free and the Q values were close to 100% within the image. The main difference between the Roemer and COMPOSER results was that the Roemer phase was subject to a slow background variation reflecting B_1_^+^ inhomogeneity, apparent as an additional closed isophase contour at arrow 6. A left‐right profile through the center of this slice showed a total phase variation of approximately 6 rad in the COMPOSER reconstruction and 11 rad in the Roemer reconstruction.

**Figure 3:**
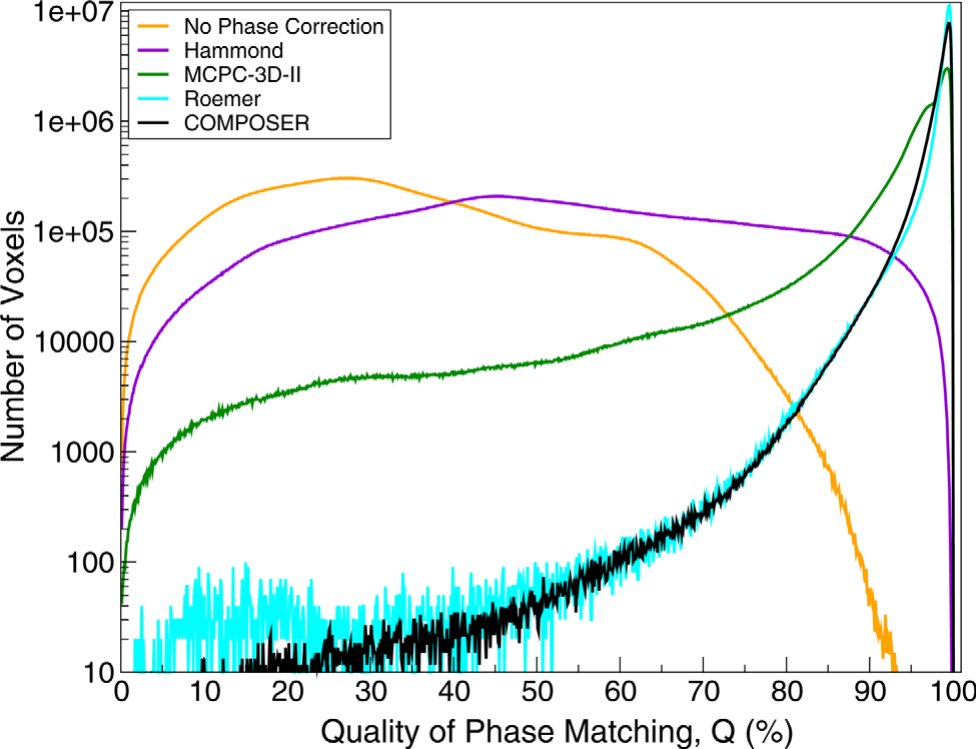
Quantitative comparison of the quality of phase matching, assessed using the quality metric Q, defined in Eq. (7). Plots are mean values over the brains of six subjects, except the Roemer method, which is for one subject. The ordinate has been scaled logarithmically to allow comparison of the relative number of voxels with poor matching.

These observations about phase‐matching quality, illustrated by isolated slices in a single subject, are confirmed by the whole‐brain quantification (Fig. 3), which was for six subjects for all methods other than Roemer (one subject). The median value of Q was 19.0 ± 0.9% with no phase correction, 50.9 ± 2.2% with Hammond, 96.9 ± 2.3% for MCPC‐3D (with PRELUDE in 2D), 99.2% for Roemer, and 98.9 ± 0.5% for COMPOSER.

COMPOSER phase images could be unwrapped without errors with PRELUDE in 2D and the Cusack method, and are shown for all subjects in Supporting Figure S2.

The origin of a small fraction of poorly matched voxels in MCPC‐3D (those to the left of the shoulder at approximately 95% in Fig. 3) is explored in Supporting Figure S3: Spatial phase unwrapping of phase images from the two TEs led to different shifts in the position of an unresolvable, open‐ended fringe line (at cyan arrows). Similarly, signal from the scalp and eyes was not consistently unwrapped because of a lack of continuity between these regions and the brain (eg, at green arrow). Likewise, the low signal in frontal areas, particularly at the second TE, led to errors in this region (discontinuity at red arrow). The phase image used in COMPOSER has a higher signal throughout because of the short TE (0.8 ms). Two wraps are visible: a well‐behaved open‐ended fringe line at the yellow arrow numbered 1), which originates in a region of the image where there is no signal (see magnitude at the corresponding position)—a legitimate occurrence in a single‐channel image; and a resolvable closed loop at the arrow numbered 2. This phase image is applied without unwrapping in the COMPOSER combination, thus avoiding potential errors from the propagation of wraps.

**Figure 4:**
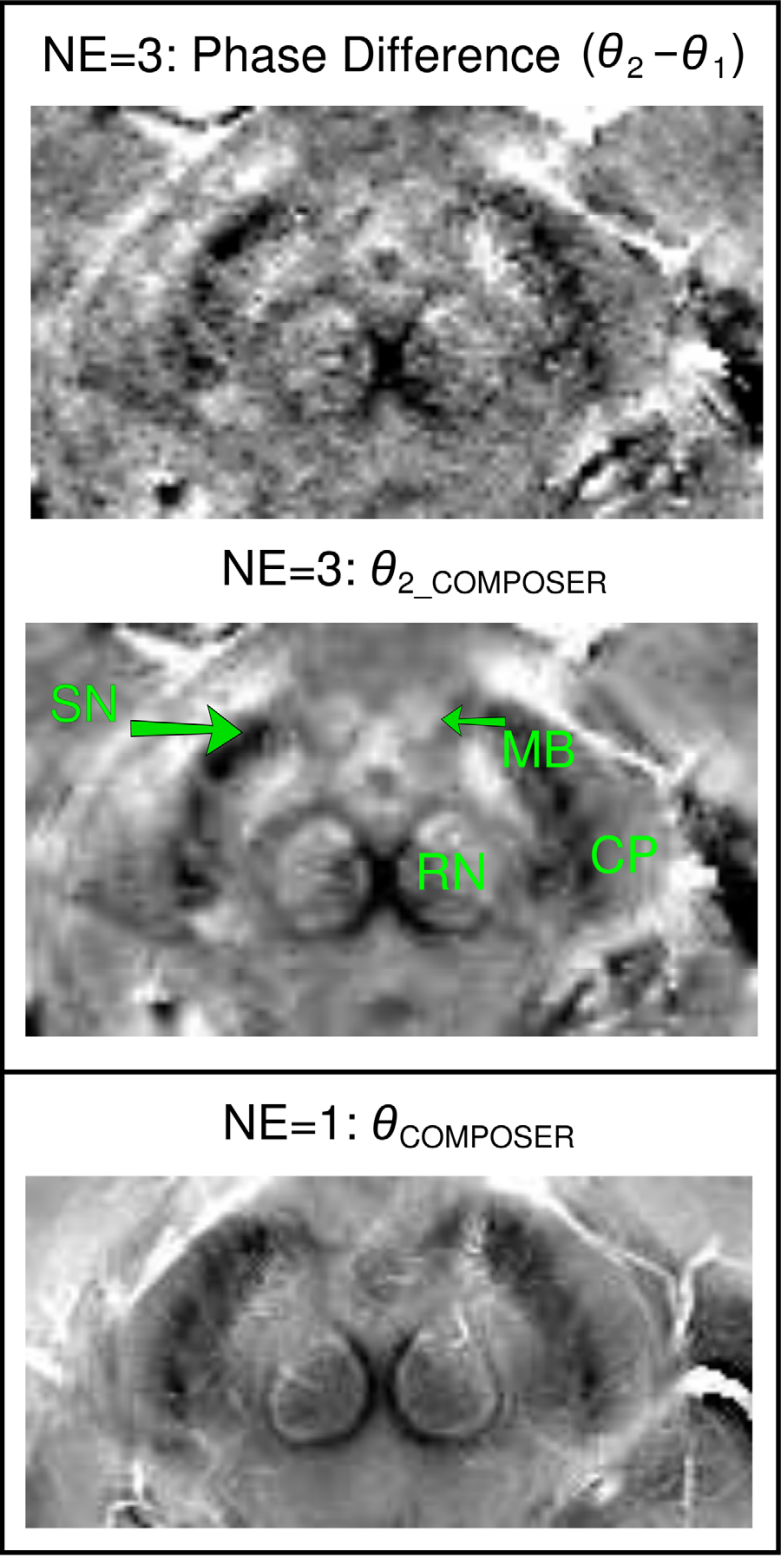
A comparison of the quality of a COMPOSER phase image and a phase difference reconstruction, illustrated in a detail of an axial midbrain slice at the level of the superior colliculus (CP, cerebral peduncles; SN, substantia nigra; RN, red nucleus; MB, mammillary body). In the multi‐echo data (NE=3; top and center), the COMPOSER reconstruction of the second echo (middle) is less noisy than the phase difference between the first and second echo (top). Additional SNR gain is made with a single‐echo acquisition with a lower bandwidth (bottom, from the protocol acquired for the group of six subjects), which was only possible with the COMPOSER approach. The images have been scaled to yield similar contrast.

COMPOSER phase images are compared with those from a conventional multi‐echo phase difference image in Figure 4. The enlargement shows structures of the ventral tegmental area and the substantia nigra pars compacta (SN), which are clearly less noisy and better resolved in the COMPOSER reconstruction. The fact that COMPOSER (and the other combination approaches tested) can be applied to single‐echo data means that higher resolution and lower bandwidth can be used with a similar TE and acquisition time, affording an improvement in the quality of imaging of this region (Fig. 4, bottom, same subject).

A comparison of signal in the image background was performed between magnitude‐only reconstructions (Supporting Figure S4, left: M_SS_ and M_rSOS_ (Eqs. (1) and (2), respectively)) and COMPOSER reconstructions, which use complex, phase‐matched data (Supporting Figure S4, right: M_COMPOSER_S_ and M_COMPOSER_W_ (Eqs. (5) and (6), respectively)). No difference is apparent if images are visualized with windowing over the full range of values (ie, between M_SS_ (Supporting Figure S4, top left) and M_COMPOSER_S_ (same figure, top right), and between M_rSOS_ (bottom left) and (bottom right)). A closer inspection of low signal values in the natural logarithms of the same images (Supporting Figure S4, central four subfigures), however, reveals reduced background in the COMPOSER reconstruction (see red arrows in figure). This impression is confirmed by noise measurements made in one subject. The background noise in M_COMPOSER_S_ was lower than in M_SS_ (64 ± 55 c.f. 217 ± 46), and lower in M_COMPOSER_W_ than M_rSOS_ (26 ± 16 c.f. 46 ± 13). This phenomenon explains the low Q values observed in the background of Figure 2; all methods using a complex sum have a lower signal in noise voxels than the (magnitude only) simple sum.

**Figure 5:**
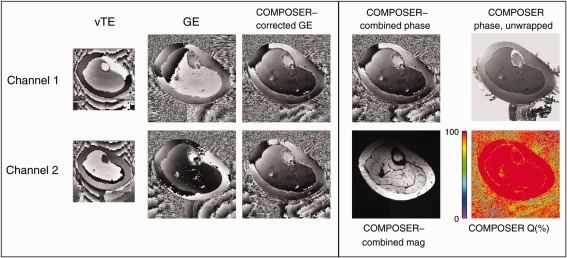
Demonstration of the quality of phase matching and reconstruction with COMPOSER with a calf coil with no volume reference. The phase images from the two proton channels (“GE” column) show little similarity before phase matching, but appear identical after phase matching. The Q values are close to 100% throughout the image. The combined phase image could be spatially unwrapped.

Supporting Figure S5 and Figure 5 show examples of phase matching with COMPOSER in the breast and calf, respectively. Both the breast and calf coils have two elements that yield very different phase images. These appear almost identical, apart from noise, after phase matching (COMPOSER‐corrected GE), with Q values close to 100% throughout the images. The combined images could be spatially unwrapped.

## DISCUSSION

We have presented a method for combining phase data from phased array coils. A coil‐wise subtraction of the phase measured in a fast, short TE reference scan from the phase in the scan of interest removes phase offsets, allowing complex data to be summed over channels. This approach was tested with coils of diverse designs in the brain, calf, and breast.

A large number of solutions to the problem of combining phase from phased array coil have been proposed in recent years. The need for appropriate methods has been stimulated by a realization of the clinical research potential of high‐resolution phase imaging with ultra‐high field MR scanners that do not have a body coil with which to perform the reconstruction described by Roemer et al 9. Some array coils have a transceive part that could be used in place of a body coil, although this will generally not be very homogeneous, and will introduce B_1_^+^ phase inhomogeneity into the combined image, an effect visible in Figure 2 and seen in other studies (eg, Fig. 1 in 43).

The comparison methods in this study have a range of features. The Hammond method 22 is computationally light but known to be imperfect distal from the object center, especially at field strengths, at which the wavelength (which is close to 12 cm in tissue at 7 T compared with approximately 30 cm at 3 T 28) is comparable to the object size. MCPC‐3D yields good phase matching and has been adopted quite widely 44, 45, 46. This method requires spatial unwrapping of the phase data, however, which is challenging because of highly inhomogeneous signal in single‐channel phase images. Regardless of the spatial unwrapping method used (PRELUDE in 2D and 3D 38, PHUN 47, CUSACK 47), we found that unwrapping led to open‐ended fringe lines in areas of low signal and errors where there was disconnected signal. These propagated into areas of higher signal, thus corrupting the combined phase. Such errors, also observed in the Hammond and the Adaptive Combined approaches, pose the risk of being misinterpreted as microbleeds or other pathologies 3, 48. COMPOSER has a computational complexity similar to the MCPC method but achieves better phase matching than MCPC‐3D (the best alternative reference‐free method tested) and equivalent to that achieved with the Roemer method (which needs a volume reference coil). B_1_^+^ inhomogeneities are present in the Roemer reconstruction. Although high‐pass filtering in SWI and QSM processing removes these, there are contexts, such as distortion correction in EPI, in which it is useful for single‐echo phase images to contain no other phase contribution than that from the B0 field 49, 50. The COMPOSER reconstruction removes B_1_^+^ variations over the object as long as the same RF excitation pulses are used for the SER and target scans. The use of different RF pulses for the two scans would not affect the quality of phase matching, but the ratio of the phases of the two would be retained in the combined image. As in the Roemer phase images in the brain, no artifacts were observed with COMPOSER in the brain, calf, or breast with coils of quite different designs.

Multi‐echo phase imaging has SNR advantages over single‐echo phase imaging and opens up the possibility to combine phase images using the temporal evolution of the phase (eg, using singular value decomposition 19). However, the total volume of data, gradient switching demands, dB/dt limitations and short T2* star values at UHF (in some tissues in particular 51) may contribute to multi‐echo imaging being undesirable in some contexts. For this reason, we focused on approaches that do not need a reference coil measurement and that can be applied to single‐echo acquisitions.

The subtraction of SER phase in COMPOSER could potentially decrease phase contrast, increase noise, and introduce errors into the scan being reconstructed. In practice, however, the very short TE and low resolution of the SER scan mean that these phase images contain minimal susceptibility‐related phase and have high SNR, even in regions affected by signal loss in the target acquisition. Both of these favorable characteristics of SER images are enhanced by the smoothing involved in the interpolation during image coregistration. As a consequence, there is no noticeable reduction in phase contrast or increase in noise as a result of the phase‐matching process, in contrast to conventional phase difference imaging.

Phase‐matched, multi‐channel data can also be used as the basis for an improved magnitude reconstruction. Magnitude‐only reconstructions such as the root sum‐of‐squares add the noise from each coil. In contrast, in complex sums, noise tends to cancel, as the complex signals point in all directions with equal probability 52. Our findings are in line with that understanding: Magnitude reconstructions from complex data (M__COMPOSER_S_ and M__COMPOSER_W_) had lower signal in voxels in the background and veins. This might provide an improvement in the contrast between veins and surrounding tissue in SWI, for instance. Because this effect increases with the number of coils, it is expected to become more relevant with the next generation of head and cardiac arrays with even larger numbers of elements 53.

The use of the metric Q has allowed the quality of phase matching to be assessed without knowledge of the ground truth phase, but is subject to two limitations. First, Q should only be close to 100% in voxels with high SNR. For low SNR voxels (eg, background), noise cancels in the complex reconstructions and a low value of Q is desirable. A magnitude threshold was applied to exclude these voxels from our analysis. Second, Q values do not reflect some desirable properties of phase images such as contrast. For instance, setting the phase of all voxels to zero in all channels would lead to Q values of 100%, but eliminate phase information. Phase reconstruction methods that are likely to result in dramatically different contrast (because they are dependent on high‐pass filtering, for instance) should be compared on the basis of phase contrast and the phase‐matching metric Q. Finally, we have chosen to define Q as the ratio of M_[METHOD]_S/_M_SS_, to enable the comparison with previous work 26. A less intuitive but equally informative measure would be M_[METHOD]_W_/M_rSOS_. The use of this index would not affect the relative performance of the methods tested nor the conclusions reached in this study.

The SER scan should have a TE that is short compared with the T2* of the tissues being imaged and be free of artifacts. If coregistration to the target scan is required (rather than simple linear interpolation, for instance), it must be well resolved so that coregistration can be accurately performed. The use of low resolution is an advantage in achieving short TE and high SNR, and also in blurring the small amount of susceptibility‐related phase evolution present. Gibbs ringing in very low resolution SER acquisitions (below approximately 3 mm isotropic resolution) led to a reduction in the quality of phase matching by a few percent. A preparatory study reduced this to negligible levels by using the voxel sizes of circa 2 mm. A vTE sequence with nonselective excitation was used for the SER measurements in this study. In the brain, the median quality of phase matching was close to 99% (98.9 ± 0.5%). A small number of voxels had reduced values (Q circa 95%) as a result of wrap‐around of a signal intensity from ventral regions in the SER scan into the top slices. Although COMPOSER outperformed the other reference‐free reconstruction approaches significantly, slight further improvement (to parity with Roemer) could be achieved using selective excitation or increasing the imaging slab in the head‐foot direction. Other short TE sequences could also be deployed, such as UTE 54, PETRA 55, or simply a GE scan with the shortest possible TE. Our preparatory experiments suggest that a conventional low‐resolution GE is adequate, although some reduction in phase contrast would be expected if the echo times of the SER and the target scan would be similar (eg, in imaging very short T2* species). A further limitation of this study is that, for consistency with other methods 22, 26, simple magnitude weighting (instead of magnitude squared weighting) was used in the calculation of combined phase images and that noise correlation between channels was neglected in the calculation of both combined magnitude and phase images.

In addition to removing B_1_^−^, which is essential to allow the combination of the channels without destructive interference, COMPOSER removes B_1_^+^, under the proviso that the same pulses are used for the SER and target scans. The use of different pulses would lead to the residual of B_1_^+^‐related phase, which, being common to all channels, would not compromise the quality of the combination but might be undesirable in some contexts.

In conclusion, we have presented a simple method for the combination of phase data from coil arrays. A fast reference scan with a short TE is used to measure the phase offset of each coil. Subtracting this from the corresponding phase values of a (generally high resolution) scan of interest removes both B_1_^+^ and B_1_^−^ inhomogeneities, matching the phases from the coils and allowing the complex signals to be combined. This method (COMPOSER) requires no reference coil, making it feasible for use with all phased arrays, including parallel transmit arrays. It is compatible with parallel imaging, requires no phase unwrapping, fitting or iterative steps, and provides phase matching that is superior to that achieved with the other reference coil–free approaches tested.

## ACKNOWLEDGMENTS

This study was funded by the Austrian Science Fund (FWF) project KLI264.

## Supporting information


**Supporting Figure S1**: A schematic illustration of phase matching by removing phase offsets and the rationale behind the quality index, Q. Left: Four complex vectors representing the signals from four channels of a coil array have different phases because each is subject to a different phase offset. Middle: With no phase correction, the magnitude of the resultant (the blue vector) is small, and Q, the ratio of the resultant M_No_Correction_S to the sum of the individual magnitudes M_SS, is correspondingly low (34%). Right: Subtracting the phase offset $\theta_{0,l}$ from each raw signal (dashed red vectors) removes the channel‐dependent phase, leaving only the susceptibility‐related contribution, which is the same for each channel other than noise. The phases of the individual signals (black vectors) are similar, and the ratio Q is close to 100%.
**Supporting Figure S2**: COMPOSER phase images for all subjects, spatially unwrapped with the Cusack method.
**Supporting Figure S3**: Comparison of a phase offset map calculated with the MCPC‐3D method and the equivalent short‐echo‐time reference used in COMPOSER. The MCPC‐3D phase offset map for this channel contains errors due to low signal in the magnitude at the second TE (red arrow). An open‐ended fringe line was propagated to different positions in the two contributing echoes (blue arrows), and a discontinuity in signal in the scalp led to an erroneous phase value in the scalp on the left‐hand side of the image. Although mostly constrained to regions of low signal, these effects constitute the small residual errors apparent in Fig. 3. No such errors are apparent in the short TE phase reference image used in COMPOSER. The shorter TE yields high and continuous signal, and the absence of the need to unwrap the phase removes errors from that process. Wraps marked 1 and 2 in the COMPOSER SER phase image are well behaved and described in the text.
**Supporting Figure S4**: A comparison of background noise in magnitude reconstructions (one slice of a high‐resolution magnitude image from the main study). Reconstruction methods that use only magnitude information (top and bottom left) show higher levels of background noise than those in which data are complex combined using COMPOSER. In the central four (2 × 2) images, the natural logarithm of values has emphasized the noise features. All images are scaled between 0 and 8. Red arrows highlight the lower background signal in the COMPOSER reconstructions.
**Supporting Figure S5**: Demonstration of the quality of phase matching with COMPOSER with a breast coil, no volume reference, and little overlap between the elements. The phase images from the two coils (“GE” column) show little similarity before phase matching, but appear identical after phase matching with COMPOSER. The combined phase image could be spatially unwrapped.
