# Supplementary material for: Combining phase images from array coils using a short echo time reference scan (COMPOSER)
Source: Magn Reson Med. 2015 Dec 29;77(1):318–27. doi: 10.1002/mrm.26093 (PMC5217082; doi:10.1002/mrm.26093)
Supplement: Supplementary file 1 — Supporting Figure S1: A schematic illustration of phase matching by removing phase offsets and the rationale behind the quality index, Q. Left: Four complex vectors representing the signals from four channels of a coil array have different phases because each is subject to a different phase offset. Middle: With no phase correction, the magnitude of the resultant (the blue vector) is small, and Q, the ratio of the resultant M_No_Correction_S to the sum of the individual magnitudes M_SS, is correspondingly low (34%). Right: Subtracting the phase offset θ0,l from each raw signal (dashed red vectors) removes the channel‐dependent phase, leaving only the susceptibility‐related contribution, which is the same for each channel other than noise. The phases of the individual signals (black vectors) are similar, and the ratio Q is close to 100%. Supporting Figure S2: COMPOSER phase images for all subjects, spatially unwrapped with the Cusack method. Supporting Figure S3: Comparison of a phase offset map calculated with the MCPC‐3D method and the equivalent short‐echo‐time reference used in COMPOSER. The MCPC‐3D phase offset map for this channel contains errors due to low signal in the magnitude at the second TE (red arrow). An open‐ended fringe line was propagated to different positions in the two contributing echoes (blue arrows), and a discontinuity in signal in the scalp led to an erroneous phase value in the scalp on the left‐hand side of the image. Although mostly constrained to regions of low signal, these effects constitute the small residual errors apparent in Fig. 3. No such errors are apparent in the short TE phase reference image used in COMPOSER. The shorter TE yields high and continuous signal, and the absence of the need to unwrap the phase removes errors from that process. Wraps marked 1 and 2 in the COMPOSER SER phase image are well behaved and described in the text. Supporting Figure S4: A comparison of background noise in magnitude reconstructions (one slice [file MRM-77-318-s001.docx]

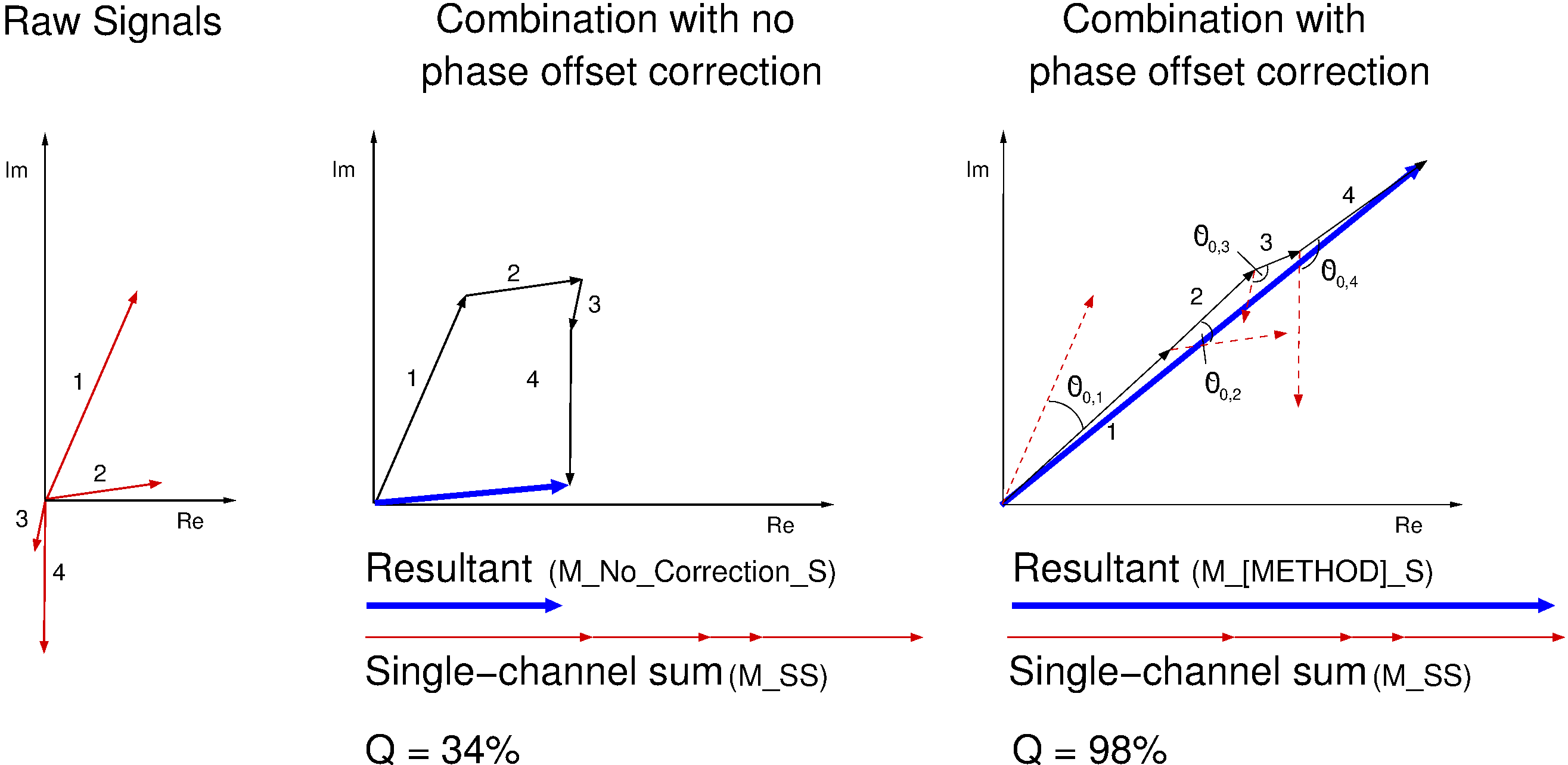


Supporting Figure S1: A schematic illustration of phase matching by removing phase offsets and the rationale behind the quality index, Q. Left: four complex vectors representing the signals from four channels of a coil array have different phases because each is subject to a different phase offset. Middle: with no phase correction, the magnitude of the resultant (the blue vector) is small and Q, the ratio of the resultant, M_No_Correction_S to the sum of the individual magnitudes, M_SS, is correspondingly low (34%). Right: subtracting the phase offset ****from each raw signal (dashed red vectors) removes channel-dependent phase, leaving only the susceptibility-related contribution, which is the same for each channel other than noise. The phases of the individual signals (black vectors) are similar and the ratio Q is close to 100%.


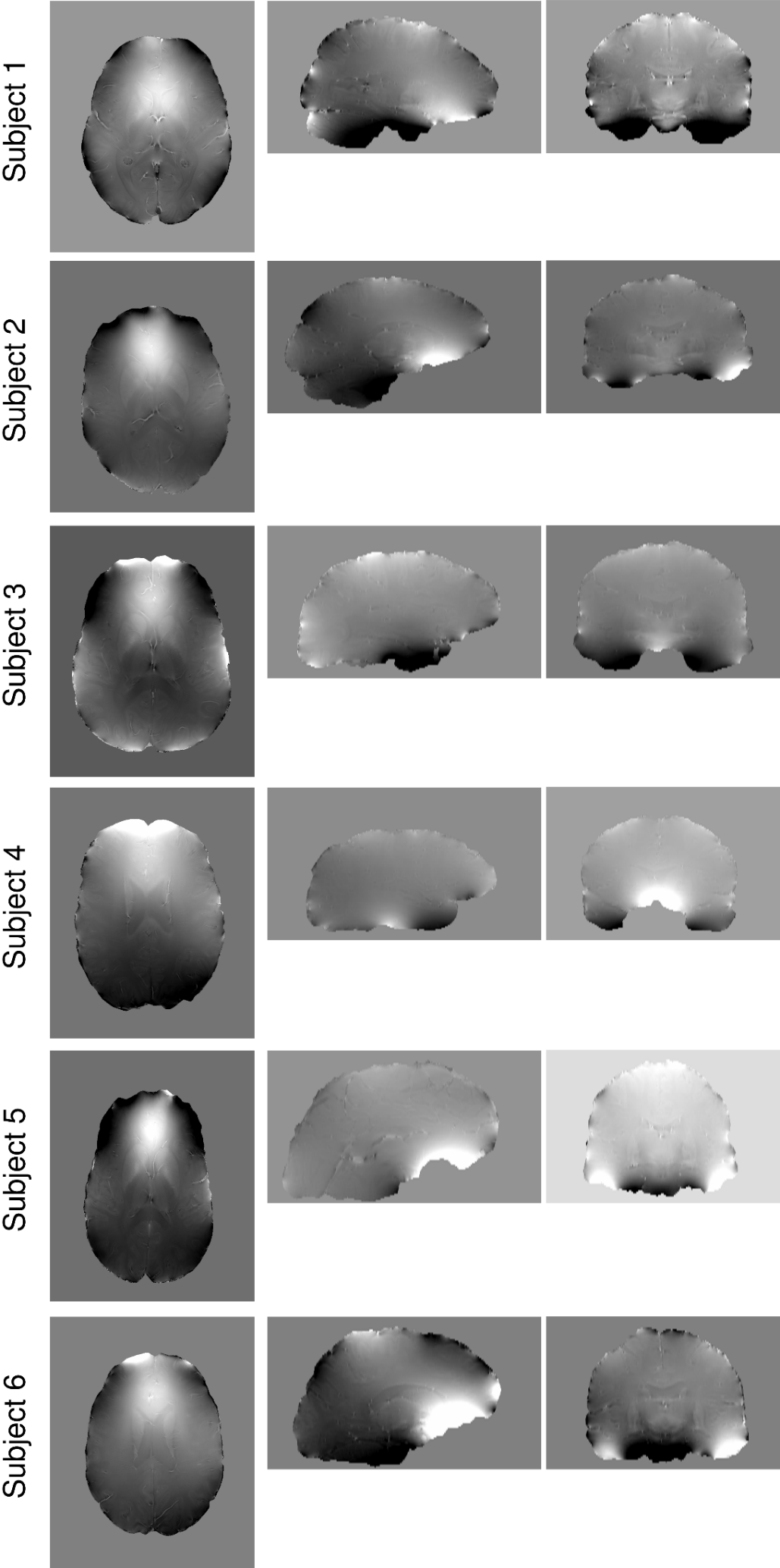


Supporting Figure S2: COMPOSER phase images for all subjects, spatially unwrapped with the Cusack method.


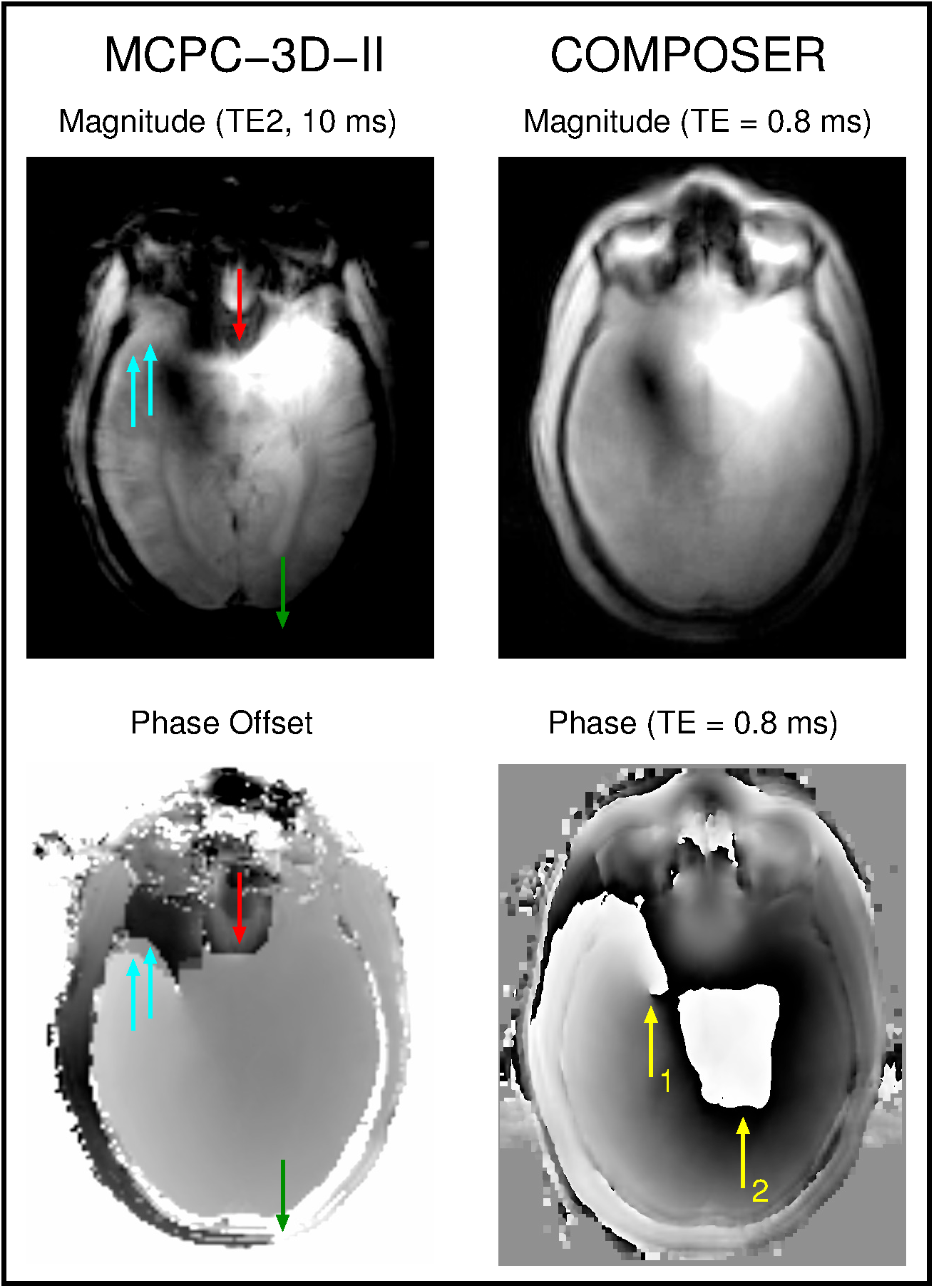


Supporting Figure S3: Comparison of a phase offset map calculated with the MCPC-3D method and the equivalent short echo-time reference used in COMPOSER. The MCPC-3D phase offset map for this channel has errors due to low signal in the magnitude at the second TE (red arrow). An open-ended fringe line was propagated to different positions in the two contributing echoes (blue arrows) and a discontinuity in signal in the scalp led to erroneous phase value in the scalp on the left hand side of the image. Although mostly constrained to regions of low signal, these effects constitute the small residual errors apparent in Figure 3. No such errors are apparent in the short TE phase reference image used in COMPOSER. The shorter TE yields high and continuous signal, and the absence of the need to unwrap phase removes errors from that process. Wraps marked 1 and 2 in the COMPOSER SER phase image are well behaved and described in the text.


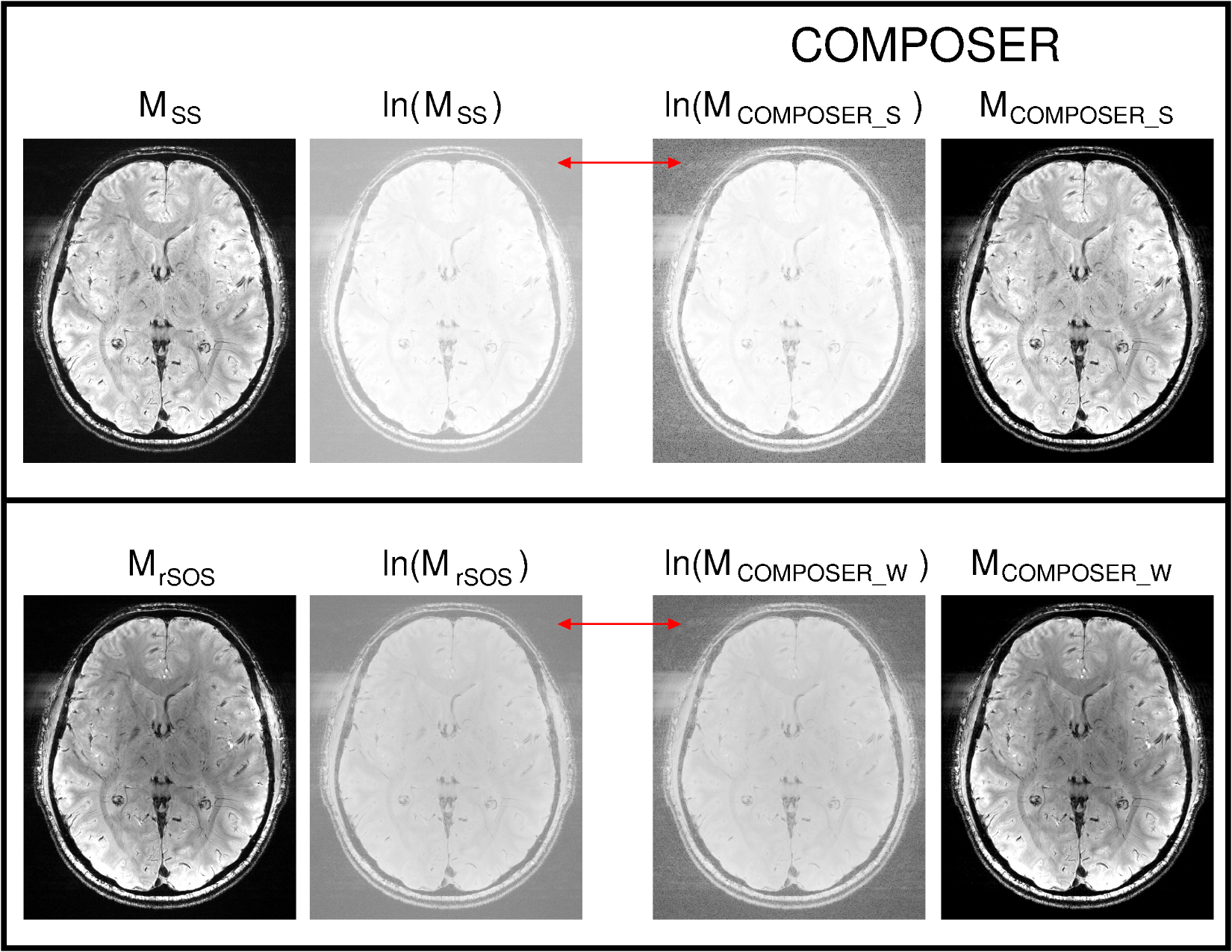


Supporting Figure S4: A comparison of background noise in magnitude reconstructions (one slice of a high resolution magnitude image from the main study). Reconstruction methods which only use magnitude information (top and bottom left) show higher levels of background noise than those in which data is complex combined using COMPOSER. In the central four (2 × 2) images the natural logarithm of values has been taken to emphasize noise features. All images are scaled between 0 and 8. Red arrows highlight lower background signal in the COMPOSER reconstructions.


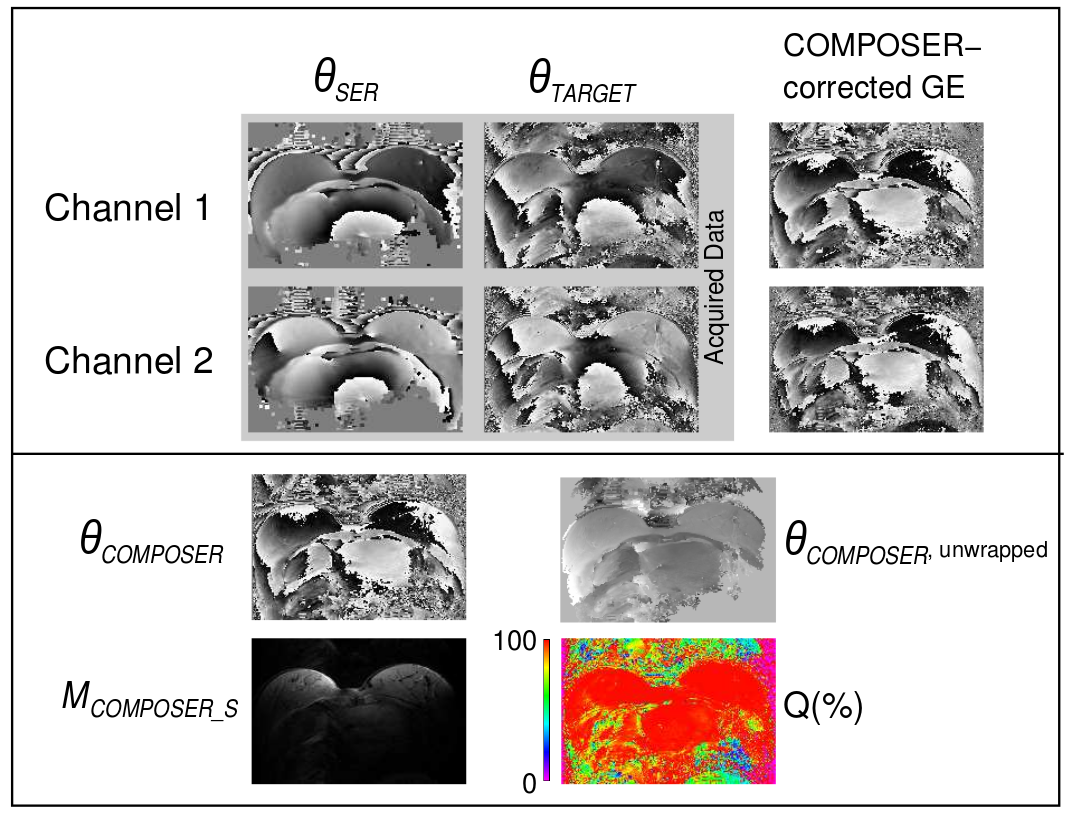


Supporting Figure S5: Demonstration of the quality of phase matching with COMPOSER with a breast coil with no volume reference and little overlap between the elements. The phase images from the two coils (“GE” column) show little similarity before phase matching, but appear identical after phase matching with COMPOSER. The combined phase image could be spatially unwrapped.
